# Antagonism at combined effects of chemical fertilizers and carbamate insecticides on the rice-field N_2_-fixing cyanobacterium *Cylindrospermum* sp. *in vitro*

**DOI:** 10.2478/intox-2014-0001

**Published:** 2014-07-16

**Authors:** Rabindra N. Padhy, Nabakishore Nayak, Shakti Rath

**Affiliations:** 1 Department of Botany, BJB Autonomous College, Bhubaneswar 751014, Odisha, India; 2 Central Research Laboratory, IMS & Sum Hospital, Siksha ‘O’ Anusandhan University, Bhubaneswar-751003, Odisha, India

**Keywords:** cyanobacterium, *Cylindrospermum*, insecticides, chemical fertilizers, toxicity, antagonism

## Abstract

Effects of chemical fertilizers (urea, super phosphate and potash) on toxicities of two carbamate insecticides, carbaryl and carbofuran, individually to the N_2_-fixing cyanobacterium, *Cylindrospermum* sp. were studied *in vitro* at partially lethal levels (below highest permissive concentrations) of each insecticide. The average number of vegetative cells between two polar heterocysts was 16.3 in control cultures, while the mean value of filament length increased in the presence of chemical fertilizers, individually. Urea at the 10 ppm level was growth stimulatory and at the 50 ppm level it was growth inhibitory in control cultures, while at 100 ppm it was antagonistic, *i.e.* toxicity-enhancing along with carbaryl, individually to the cyanobacterium, antagonism was recorded. Urea at 50 ppm had toxicity reducing effect with carbaryl or carbofuran. At 100 and 250 ppm carbofuran levels, 50 ppm urea only had a progressive growth enhancing effect, which was marked well at 250 ppm carbofuran level, a situation of synergism. Super phosphate at the 10 ppm level only was growth promoting in control cultures, but it was antagonistic at its higher levels (50 and 100 ppm) along with both insecticides, individually. Potash (100, 200, 300 and 400 ppm) reduced toxicity due to carbaryl 20 and carbofuran 250 ppm levels, but potash was antagonistic at the other insecticide levels. The data clearly showed that the chemical fertilizers used were antagonistic with both the insecticides during toxicity to *Cylindrospermum* sp.

## Introduction

Global food security is a matter of concern today with multiple food demands by rising populations and the decrease of farm-land area for grain/food production. Since the start of the ‘green revolution’ in the 1960s, commercial agriculture has been using high-yielding varieties of crops, for which a constant input of agrochemicals – chemical fertilizers and pesticides are needed. However, the addition of any agrochemical to an agro-ecosystem continually each year, for example a patch of rice field, affects the dynamic equilibrium of the soil environment (Bambaradeniya & Amerasinghe, 2003). Non-target useful flora and fauna are heavily affected. Soil being the leitmotiv of all vital events in an agricultural environment, replenishments of nutrients through bioremediation of organic materials, detoxication of pollutants, fermentation, putrefaction and a few more associated microbial processes (Hill & Wright, 1978; Zancan *et al.*, 2006), as well as the availability of minerals from the smooth running of several biogeochemical cycles in coordination by microbes of the detritus layer, are jeopardized (Roger, 1995). Specifically, a temporary prevention / moratorium of natural additions of organics and nitrogenous compounds by microbes, including N_2_-fixers, occur by unsustainable additions of chemical fertilizers (Mohammadi & Sohrabi, 2012), leading to form-sifting of crop-soil. Soil compaction may occur eventually, leading to associated changes in its permeability (Ankeny *et al.*, 1990), resulting in denitrification. Anyway, soil fertility and productivity are affected by pesticides, at length (Savci, 2012); no matter, the intended crop may be well nourished for good in the commercial farm practice by added agro-chemicals in the current crop-season.

Indeed, filamentous, heterocystous N_2_-fixing cyanobacteria growing in the flooded rice soils along with rice crop are known to maintain soil health by the addition of N-compounds, and that from time immemorial, when the use of urea was unknown (Swaminathan 1984; Prasanna *et al.*, 2013). The production of urea industrially and its use in rice agriculture landed at blasting diminution of soil N_2_-fixing cyanobacteria (Patnaik & Singh, 1978; Othma *et al.*, 2013), as urea is a contrivance to the heterocyst (the site of N_2_-fixation) differentiation and activity of the nitrogenase enzyme complex (Bothe & Eisbrenner, 1981). Yet there is a logistic need for the use of chemical N-fertilizers to obtain maximal rice yield in commercial agriculture. Non-nitrogenous fertilizers, superphosphate and potash, are known to promote the growth of cyanobacteria during limited field uses (Singh, 1975; Irisarri *et al.*, 2007). Further, intensive rice farming needs the application of a chemical N-fertilizer once during the growing stage of the crop and once more during the flowering stage, since the application of split doses of a chemical fertilizer is always a desirable practice. Concomitantly, the crop needs constant protection from intruding pests.

Specifically, adverse effects of pesticides on N_2_-fixing soil cyanobacteria *in vitro* have been well documented, with limited *in vivo* studies (Padhy, 1985a; Pipe, 1992; Prasad & Vaisampayana, 1994; Subramanian *et al.*, 1994; Paul & Clarke, 1996; Koenig 2001; Whitton & Potts, 2002; Aktar *et al.*, 2009). In a German crop field, a combined application of pesticides and chemical fertilizers had suppressed the growth of cyanobacteria by competitive development of other algae (Sauthoff & Oesterreicher, 1994). Furthermore, in Philippine rice soils applications of N or P or K chemical fertilizers suppressed the growth of cyanobacteria and acetylene reduction (N_2_-fixation *in situ*), when grazers belonging to Gastropoda and Ostrapoda were suppressed by use of insecticides (Roger, 1995; Aktar *et al.,* 2009). Moreover, statistical figures of total consumption of individual insecticides, fungicides and herbicides for productions of food and cash crops are uncomfortably high in each country. For instance in Indian agriculture, 107.6 and 554.2 metric tons technical grades of carbaryl and carbofuran, respectively were used on average in 5 years, between 2005 and 2010 (Anonymous, 2011). Further, the most common rice-pests, the brown plant hopper (*Nilaparvata lugens*), green leaf hopper (*Nephothettix virescens*), stem borers (*Typhorhiza incertulas, Scirpophaga innotata, S. incertulas, Chilo suppressalis*), the whorl maggot (*Hydrellia philippia*), water weevil (*Lissorhoptrus oryzophilus*) and a few more are controlled by the two broad spectrum systemic carbamate insecticides, carbaryl and carbofuran.

Maintaining a water-logging condition before puddling is not a problem in low land rice fields, as in many parts of Vietanam and other Asian countries. N_2_-fixing cyanobacteria are grown in rice fields before puddling without the use of any chemical N-fertilizer and are mixed with soil by puddling as biofertilizers, for a cost-effective, sustainable and natural N-nutrition of rice (Padhy, 1985b; Whitton *et al.*, 1988; Prasanna *et al.*, 2013). As usual, super phosphate and potash are used as other P and K chemical fertilizers during the growing stage of cyanobacterial biofertilizers.

In this perspective, monitoring three commonly used chemical fertilizers (urea, super phosphate and potash), individually along with two commonly used insecticides (carbaryl and carbofuran), individually on the growth of a rice field N_2_-fixing cyanobacterium, *Cylindrospermum* sp. is pursued herein, as cyanobacterial biofertilizers are considered to be eco-friendly and agro-friendly aids in rice-farming, as well as both pesticides and chemical fertilizers are routinely used in commercial rice agriculture.

## Materials and methods

The filamentous heterocystous N_2_-fixing cyanobacterium (blue-green alga) *Cylindrospermum* sp. (PK Singh strain) was originally isolated from rice-fields of Cuttack, Odisha into axenic cultures. The cyanobacterium is basically planktonic and has two polar heterocysts. It forms fine suspension of hand-shaken batch cultures. The cyanobacterium was grown in modified Chu-10 medium under the N_2_-fixing condition with the following compositions (ppm): MgSO_4_.7H_2_O, 25; Na_2_CO_3_, 20; Na_2_SiO_3_.5H_2_O, 44; CaCl_2_.2H_2_O, 55.5; K_2_HPO_4_, 10; ferric citrate, 3.5; citric acid, 3.5. Trace elements were (ppm): H_2_BO_3_, 0.5; MnSO_4_.7H_2_O, 0.5; ZnSO_4_.7H_2_O, 0.05; CuSO_4_.5H_2_O, 0.02; MoO_3_, 0.01; CoCI_2_.6H_2_O, 0.04 in a stock solution from which an aliquot of 1 mL was added to 1 L of growth medium, designated as C-N medium. In place of CaCl_2_.2H_2_O, 232 ppm of Ca(NO_3_)_2_ per 1 L was added for the nitrate supplemented Chu-10 medium, designated as C+N medium. Growth was measured as OD_660_ of cultures on day-1 and day-8 of the inoculation. Commercial grades of chemical fertilizers, urea, super phosphate and potash, as well as two insecticides carbaryl and carbofuran were used. The chemical names of the pesticides used are carbaryl: 1-naphthyl methyl carbamate and carbofuran: 2,3-dyhydro-2-2-dimethylbenzofuraan-7yl methyl-carbamate; their commercial formulations used with field recommendation doses were Sevin 50W (3.5 kg/ha) and Furadan 3G (12.5 kg/ha), respectively. Growth experiments were conducted in culture tubes with 10 mL of final volumes of cultures, having mixtures of a chemical fertilizer and 0.5 mL inoculum of the cyanobacterium at the actively growing (7 to 10 days old) stage with 3.5x10^4^ to 3.8×10^4^ cells/mL. Measurements of filament length were done by an ocular micrometer. The pooled data of the second repeated experiment were computed for statistical significance.

## Results

At 10 ppm urea (46% N), 10 ppm super phosphate (P_2_O_5,_ 16% P) and 100 ppm potash (K_2_O, 60% K) individually, optimum growth patterns of the cyanobacterium were recorded. The respective higher doses (50 ppm urea, 50 ppm super phosphate and 200 ppm potash) were mildly toxic to the cyanobacterium. Average numbers of vegetative cells between two polar heterocysts of random filaments and average values of filament length of cultures with urea (10 and 50 ppm), super phosphate (10 and 50 ppm) and potash (100 and 200 ppm), individually, are presented (Table 1). Filaments were longer in cultures with fertilizers. The ratio of heterocysts to vegetative cells in a filament decreased with each chemical fertilizer and the value was the lowest with urea: 58.5±7.3 µm or 59.7±8.8 µm. A longer filament would have a larger number of vegetative cells between the two polar heterocysts of the filament, and consequently a decreased ‘heterocyst to vegetative cell ratio’. Moreover, filaments were longer in the presence of other two chemical fertilizers used individually in C-N medium, since the differentiation of new heterocysts was normal (Table 1). Pearsonian *r* (correlation coefficient) values between the number of vegetative cells between heterocysts and filament length were below +0.95 (*r*=0.9237) for both control and treated cultures (Table 1), indicating no-effect of the chemical fertilizers super phosphate or potash on heterocyst differentiation. This indicated that the filament length increased by the increase of cell size, nonetheless the number of cells in a filament increased, as expected. Growth enhancements, no-observed effective concentration (NOEC), minimum inhibitory concentration (MIC), highest permissive concentration (HPC) and lethal concentration_100_ (LC_100_) were determined in the presence of individual insecticides in the growing media (Table 2). Liquid cultures at LC_100_ levels were centrifuged for 10 min at 5,000 rpm and pellets were grown in fresh pesticide-free medium for 10 days; when no increase in turbidity was noticed, the LC_100_ level was confirmed. Chemical fertilizers and insecticides interacted individually at partial lethal ranges, below the highest HPCs of both insecticides.

**Table 1:** Filament length and distances as number of vegetative cells of the two polar heterocysts of *Cylindrospermum* sp. In the presence of chemical fertilizers in an N_2_-fixing medium^a^.

Chemical fertilizers (ppm)	Filament length (µm)	Number of vegetative cells in a filament
Control	47.8 ± 8.7	16.3
Urea (10)	58.5 ± 7.3	22.4
Urea (50)	59.7 ± 8.8	22.4
Superphosphate (10)	51.3 ± 6.7	18.1
Superphosphate (50)	52.1 ± 7.5	17.3
Potash (100)	52.8 ± 6.5	17.6
Potash (200)	53.0 ± 7.0	18.8

a Each value is an average of 10 observations. From values of filament length and numbers of vegetative cells in filament from control and fertilizer treated cultures, Pearsonian r (correlation coefficient) computed was 0.9237, a value less than 0.95. Computation of heterocyst percentage in terminal heterocystous filaments such as of *Cylindrospermum* becomes ambiguous, so it was not done.

**Table 2:** Toxic ranges of *Cylindrospermum* sp. due to selected insecticides in liquid and solid media (ppm)^a^.

	Liquid cultures						Agar media	
			Partial lethal range					
Insecticides	Medium type	Growth enhancing conc.	NOEC	MIC	HPC	LC_100_	HPC	LC_100_
Carbaryl	C-N	NO	10	20	80	100	80	100
	C+N	NO	10	20	100	120	80	100
Carbofuran	C-N	25	50	100	1500	2000	500	600
	C+N	25	50	100	2000	3000	500	600

a NO = no growth enhancing dose was observed in standard inoculums at or below 3.5×10^4^ cells per mL. NOEC = no-observed effective concentration. MIC = minimum inhibitory concentration. HPC = highest permissive concentration. LC_100_ = lethal concentration 100.

### Urea

Urea levels used were 0, 10, 50 and 100 ppm; carbaryl levels used were 0, 20, 40 and 70 ppm; carbofuran levels used were 0, 25, 100 and 250 ppm. *Cylindrospermum* sp. was grown in C-N medium, along with urea and carbaryl or carbofuran. The level of 10 ppm urea alone caused optimum growth at all the levels of carbaryl or carbofuran used**.** The level of 50 ppm urea caused a better growth of the cyanobacterium at 20 ppm carbaryl. The growth patterns at 50 ppm urea and 40 ppm carbaryl were still better than that in the presence of the same dose of urea and 0 ppm carbaryl, but this situation was antagonistic as growth was less than 20 ppm carbaryl and 50 ppm urea. The dose of 25 ppm carbofuran was growth enhancing for the cyanobacterium, and the level 50 ppm urea caused growth retardation both at 0 and 25 ppm carbofuran. At 100 and 250 ppm carbofuran levels, 50 ppm urea had a progressive growth enhancing effect, which was marked well at 250 ppm carbofuran level, a situation of synergism. Thus, it could be concluded that urea at 50 ppm had a reducing effect on toxicity of carbaryl or carbofuran (Table 3). The Pearsonian *r* values across four gradations of the insecticides individually with one dose of urea (row-wise) as well as four gradation of urea with one dose of insecticide (column-wise) are presented (Table 3).

**Table 3:** Effect of urea on toxicity of carbaryl and carbofuran to *Cylindrospermum* sp.

Carbaryl (ppm)	Urea (ppm)					
	0	10	50	100	Mean	*r* value
0	0.166	0.28	0.1	0.05	0.149	−0.71
20	0.138	0.176	0.223	0.125	0.165	−0.18
40	0.043	0.078	0.181	0.033	0.084	−0.02
70	0.043	0.07	0.07	0.071	0.063	+0.61
Mean	0.097	0.151	0.143	0.07	0.115	
*r* value	−0.9	−0.91	−0.32	−0.07		
**CD**	**Carbaryl mean**	**Urea mean**	**Interaction**			
5%	0.013	0.013	0.026			
1%	0.017	0.017	0.035			

**Carbofuran (ppm)**	**Urea (ppm)**					
	**0**	**10**	**50**	**100**	**Mean**	***r* value**

0	0.286	0.37	0.26	0.01	0.231	−0.91
25	0.3	0.34	0.223	0.015	0.222	−0.96
100	0.256	0.296	0.293	0.043	0.222	−0.82
250	0.07	0.08	0.246	0.07	0.119	+0.12
Mean	0.228	0.274	0.258	0.034	0.198	
*r* value	−0.96	−0.98	+0.05	+0.98		
**CD**	**Carbofuran mean**	**Urea mean**	**Interaction**			
5%	0.0036	0.0036	0.0073			
1%	0.0049	0.0049	0.0098			

Note: OD on zero day = 0.01 for carbaryl, 0.025 for carbofuran. Data presented are averages of triplicate cultures. 25 ppm carbofuran was the growth enhancing dose. CD, critical difference.

### Super phosphate

In the absence or presence of any dose used of either insecticide, only the level of 10 ppm super phosphate caused optimum growth pattern of the cyanobacterium. In the absence of an insecticide, 50 or 100 ppm super phosphate caused progressive toxicity. Both these levels of super phosphate were found not to reduce the toxicity of any level of either insecticide, rather toxicities were found to be increased (Table 4). It can be concluded that except the very low dose (10 ppm), super phosphate increased the toxicity of both insecticides individually to the cyanobacterium; the *r* values were mostly strongly negative, suggesting antagonism (Table 4).

**Table 4:** Effect of super phosphate on toxicity of carbaryl and carbofuran to *Cylindrospermum* sp.

Carbaryl (ppm)	Super phosphate (ppm)					
	0	10	50	100	Mean	*r* value
0	0.166	0.178	0.1	0.0316	0.119	−0.98
20	0.138	0.143	0.1	0.04	0.105	−0.98
40	0.043	0.031	0.043	0.038	0.039	−0.07
70	0.043	0.031	0.043	0.035	0.038	−0.14
Mean	0.097	0.071	0.036	0.097	0.075	
*r* value	−0.90	−0.9	−0.87	+0.21		
**CD**	**Carbaryl mean**	**Super phosphate mean**	**Interaction**			
5%	0.0026	0.0026	0.005			
1%	0.0035	0.0035	0.0068			

**Carbofuran (ppm)**	**Super phosphate (ppm)**					
	**0**	**10**	**50**	**100**	**Mean**	***r* value**

0	0.286	0.336	0.145	0.03	0.199	−0.96
25	0.3	0.33	0.19	0.015	0.208	−0.98
100	0.26	0.28	0.196	0.026	0.19	−0.97
250	0.07	0.076	0.075	0.02	0.06	−0.85
Mean	0.229	0.255	0.151	0.022	0.164	
*r* value	−0.96	−0.98	−0.72	−0.24		
**CD**	**Carbofuran mean**	**Super phosphate mean**	**Interaction**			
5%	0.0032	0.0032	0.0065			
1%	0.0043	0.0043	0.008			

Note: OD on zero day = 0.01 for carbaryl, 0.025 for carbofuran. Data presented are averages of triplicate cultures. 25 ppm carbofuran was the growth enhancing dose.

### Potash

Cultures without any insecticide or 25 ppm carbofuran (the growth enhancing dose) had optimum growth patterns at 100 ppm potash. The levels of 100 and 200 ppm potash apparently reduced the toxicity due to carbaryl at 40 and 70 ppm levels. The doses 200 and 300 ppm potash caused enhancements in toxicity in the absence or presence of all the levels of carbaryl used, conforming antagonism, which is supported by negative *r*-values at each level (Table 5). It could be concluded that lower doses (100 and 200 ppm) or higher doses (200 to 400 ppm) of potash were progressively toxic at 25 and 100 ppm carbofuran. At 250 ppm carbofuran both 200 and 300 ppm potash caused better growth. However at each potash level used, increases of carbofuran caused increase in toxicity to cyanobacteria (Table 5). Only growth enhancement at 200 and 300 ppm levels at 250 ppm carbaryl caused mild synergism with a positive *r* value, +0.07.

**Table 5:** Effect of potash on the toxicity of carbaryl and carbofuran to *Cylindrospermum* sp.

Carbaryl (ppm)	Potash (ppm)						
	0	100	200	300	400	Mean	*r* value
0	0.208	0.226	0.186	0.153	0.07	0.169	−0.89
20	0.18	0.17	0.17	0.146	0.07	0.147	−0.85
40	0.046	0.051	0.05	0.045	0.036	0.046	−0.69
70	0.04	0.05	0.045	0.04	0.033	0.041	−0.59
Mean	0.118	0.124	0.112	0.096	0.052	0.101	
*r* value	−0.90	−0.91	−0.9	−0.89	−0.89		
**CD**	**Carbaryl mean**	**Potash mean**	**Interaction**				
5%	0.0032	0.0036	0.0074				
1%	0.0043	0.0048	0.01				

**Carbofuran (ppm)**	**Potash (ppm)**						
	**0**	**100**	**200**	**300**	**400**	**Mean**	***r* value**

0	0.23	0.3	0.213	0.193	0.09	0.205	−0.80
25	0.283	0.28	0.233	0.18	0.038	0.203	−0.92
100	0.256	0.25	0.213	0.151	0.026	0.179	−0.92
250	0.041	0.041	0.108	0.113	0.016	0.0638	+0.07
Mean	0.202	0.217	0.192	0.159	0.042	0.183	
*r* value	−0.89	−0.97	−0.93	−0.99	−0.75		
**CD**	**Carbofuran mean**	**Potash mean**	**Interaction**				
5%	0.0036	0.004	0.0078				
1%	0.0048	0.0043	0.01				

Note: OD on zero day = 0.01 for carbaryl, 0.025 for carbofuran.

Data presented are averages of triplicate cultures. 25 ppm carbofuran was the growth enhancing dose.

Pearsonian *r* values in Tables 3 to 5 presented here clearly indicated negative correlations mostly in row-wise and column-wise computations for the chemical fertilizers used against both insecticides, individually, indicating antagonism during the interaction of two types of agrochemicals, except a few occurrences of positive correlations. For example, urea at the 50 ppm level interacting with four levels of carbaryl yielded *r* =–0.32; this is a negative correlation indicating an antagonism of urea with carbaryl in causing toxicity to the cyanobacterium. Doses of a chemical fertilizer with four doses of any insecticide used, or *vice-versa,* with positive *r* values, should be regarded as an occurrence of synergism or reduction of toxicity, which is rare, as seen with carbofuran in the present set of data. Positive *r* values indicated synergism, while negative values indicated antagonism for both insecticides; but lower positive *r* values were indicative of weaker synergism than in 70 ppm carbaryl levels across increases of urea doses. The critical difference (CD) values computed for each set of interaction at both 95% and 99% levels were suggestive of antagonism for these interactions.

## Discussion

*Cylindrospermum* sp. has the heterocyst differentiation process linked to the division of the filament at the center into two, and generally heterocysts are differentiated at scarcity of fixed N-compounds. Urea in the present study did not inhibit filament division and heterocyst differentiation of *Cylindrospermum* sp. significantly, as elucidated with other genera (Singh, 1975; Pattnaik & Singh, 1978).

In the rice field, the organic matter supports an array of photosynthetic and non-photosynthetic soil organisms. The latter could interfere with the depletion of nutrition essential for the crop for which chemical fertilizer is needed yet could be pathogenic to rice. Concomitantly, the growth of cyanobacteria is affected by chemical fertilizers. Pesticides induce the coveted control of non-photosynthetic organisms and affect growth of cyanobacteria as the non-target effect. At such a pandemonium of the natural soil environment, the chemical fertilizers and pesticides individually/holistically should affect cyanobacteria in maintaining the natural soil health for cropping. This study records antagonistic as well as synergistic effects of three chemical fertilizers on toxicities of two insecticides on the cyanobacterium. This study should help assessing the sustainability of rice-soil environment under both types of agro-chemicals and help in preparation of a module of preservation of soil health with maximal rice yield, as a corollary to integrated pest management, when it is theoretically realized that agro-chemicals in intensive farming would be the cause of crop failures in the majority of fertile lands of the world around 2040 (Chrispeels & Sadava, 1994).

The recommended field dose of individual chemical fertilizers is 100 kg/ha (Roger *et al.*, 1984), and their levels used in the study were lower than the field recommended doses. The LC_100_ levels of both insecticides recorded herein were below their individual field recommendation doses: carbaryl (Sevin 50W) 3.5 kg/ha and carbofuran (Furadan 3G) 12.5 kg/ha. In the present study, except urea, the two other fertilizers had toxicity enhancing effect due to insecticides to the cyanobacterium, indicating antagonism. The individual chemical fertilizers, particularly super phosphate and potash in almost all doses and urea in higher doses were antagonistic with individual insecticides for growth, i.e. they caused more toxicity. The data presented here are statistically significant at both 95% and 99% levels, as known from CD values.

Under insecticide stresses, the process of N_2_-fixation by cyanobacteria has been repeatedly reported by several workers to be adversely affected (see Padhy, 1985a). In the present study, urea at lower doses (up to 50 ppm) served the demand of nitrogenous compounds for a healthy growth of cyanobacterium, in spite of the pesticide stress. As expected, the process of N_2_-fixation being sensitive should readily be impaired by chemicals of environmental concern, as exemplified with the insecticide parathion-methyl with this cyanobacterium (Panigrahi *et al.*, 2003).

Phosphate is stored as phosphate bodies in prokaryotic cells, which could divide for 3 or 4 generations down in P-deficiency. An addition of extra phosphate as K_2_HPO_4_ had specific toxicity reducing effect due to the herbicide 2,4-D in the N_2_-fixing cyanobacterium *Anabaenopsis raciborskii* (Das & Singh, 1977). On the contrary, higher doses of super phosphate were antagonistic with the insecticides individually, in the present study. Potash at lower doses reduced the toxicity due to insecticides individually, a finding agro-friendly to the rice field ecosystem. Chemical N, P and K fertilizers at field doses protected 9 genera of N_2_-fixing cyanobacteria including *Cylindrospermum* sp. from general herbicide toxicity, as reported (Tiwari & Kumar, 1989). Another study with *Wollea bharadwajae* and *C. licheniforme* recorded that at field doses, N, P and K chemical fertilizers had varied responses from species to species, but 20 ppm NH_4_Cl inhibited N_2_-fixation (Singh, 1975). An alteration of heterocyst spacing in an intercalary heterocystous form, *Nostoc muscorum* by NH_4_ salts, was reported (Singh *et al.*, 1994)*.* This strain being programed for heterocyst differentiation and filament doubling concomitantly, the filament in urea influence was having greater length than those from N-depleted cultures.

In conclusion, it could be stated that fertilizers and insecticides might be considered together for doses and mode of applications for an effective exploitation of these friendly soil microbes. The surface broadcast method of the use of N-fertilizers was recorded to suppress N_2_-fixation by cyanobacteria, with eventual growth and dominance of green algae, which temporarily bind-up or immobilize available nutrients, eventually affecting the crop. Therefore, the method of deep application of fertilizers was suggested in the literature (Roger, 1995). In Valencian rice fields, the treatment assays were done with phosphorus fertilization, straw incorporation, an insecticide (trichlorfon) application and inoculation of cyanobacteria that caused a moderate decrease of the population of N_2_-fixing heterocystous cyanobacteria, but the rice productivity was not affected (Leganés *et al.*, 2001). Such studies with soil samples in model ecosystems should clarify doubts that may arise out of probable antagonistic effects of organisms and chemicals in soil, despite the facts that chemical fertilizers and pesticides generate persisting non-target eco-toxic effects.
